# The potential mechanistic link between allergy and obesity development and infant formula feeding

**DOI:** 10.1186/1710-1492-10-37

**Published:** 2014-07-22

**Authors:** Bodo C Melnik

**Affiliations:** 1 Department of Dermatology, Environmental Medicine and Health Theory, University of Osnabrück, Sedanstrasse 115, DE-49090 Osnabrück, Germany

**Keywords:** Allergy, Breastfeeding, FoxP3, Infant formula, mTORC1, Obesity, Postnatal growth acceleration, Regulatory T-cell

## Abstract

This article provides a new view of the cellular mechanisms that have been proposed to explain the links between infant formula feeding and the development of atopy and obesity. Epidemiological evidence points to an allergy- and obesity-preventive effect of breastfeeding. Both allergy and obesity development have been traced back to accelerated growth early in life. The nutrient-sensitive kinase mTORC1 is the master regulator of cell growth, which is predominantly activated by amino acids. In contrast to breastfeeding, artificial infant formula feeding bears the risk of uncontrolled excessive protein intake overactivating the infant’s mTORC1 signalling pathways. Overactivated mTORC1 enhances S6K1-mediated adipocyte differentiation, but negatively regulates growth and differentiation of FoxP3^+^ regulatory T-cells (Tregs), which are deficient in atopic individuals. Thus, the “early protein hypothesis” not only explains increased mTORC1-mediated infant growth but also the development of mTORC1-driven diseases such as allergy and obesity due to a postnatal deviation from the appropriate axis of mTORC1-driven metabolic and immunologic programming. Remarkably, intake of fresh unpasteurized cow’s milk exhibits an allergy-preventive effect in farm children associated with increased FoxP3^+^ Treg numbers. In contrast to unprocessed cow’s milk, formula lacks bioactive immune-regulatory microRNAs, such as microRNA-155, which plays a major role in FoxP3 expression. Uncontrolled excessive protein supply by formula feeding associated with the absence of bioactive microRNAs and bifidobacteria in formula apparently in a synergistic way result in insufficient Treg maturation. Treg deficiency allows Th2-cell differentiation promoting the development of allergic diseases. Formula-induced mTORC1 overactivation is thus the critical mechanism that explains accelerated postnatal growth, allergy and obesity development on one aberrant pathway.

## Introduction

Allergy and obesity are common diseases of developed countries. Epidemiological evidence points to an allergy-preventive effect of breastfeeding [1,2]. The widespread use of artificial infant formula feeding is the most recent nutritional change introduced by industrialized societies a century ago, a time when milk has been misinterpreted as *just food*[3,4]. American paediatricians of the 1930’s were convinced that *it should be perfectly possible to prepare an artificial formula that meets all the nutritional requirements of a growing infant*[3,4]. Notably, formula feeding has been introduced straight into infant nutrition without any prior knowledge of *mechanistic target of rapamycin complex 1* (mTORC1) signalling, a novel field of molecular biology that evolved after the discovery of the natural mTORC1-inhibitor rapamycin. This immunosuppressive natural product of *Streptomyces hygroscopicus* was found on Easter Island (Rapa Nui) and has been characterized in 1975 [5,6]. This finding initiated modern mTOR biology that followed 45 years after the introduction of artificial formula feeding in the 1930’s.

Based on translational research, this review links recent insights into mTORC1 regulation to postnatal infant growth and aberrant developmental programming of the immune system and adipocyte differentiation.

## Review

### mTORC1: the cell’s master regulator of cell growth

In the last decade, the nutrient-sensitive kinase mTORC1 has been extensively studied and appreciated as the cell’s master regulator orchestrating cell growth, cell proliferation and autophagy [7-10]. mTORC1 senses multiple internal and external signals such as cellular energy status, the growth factors insulin and insulin-like growth factor-1 (IGF-1), and most importantly amino acid availability [7-10]. mTORC1 is activated at the lysosomal membrane in the presence of amino acids, especially by leucine and glutamine, which play a predominant role for mTORC1 activation [11-17]. Thus, there is a direct link between amino acid availability and mTORC1-driven cell growth.

### Infant weight gain and allergy risk

The milk protein content of a mammalian species highly correlates with its postnatal growth rate [18]. For instance, rats with a milk protein concentration of approx. 11 g/dL double birth weight after 4 days, cats and dogs with 8 to 9 g/dL after 10 days, cows with 3.5 g/dL after 40 days, and humans with 1.2 g/dL after 180 days, respectively [18]. Thus, *Homo sapiens* exhibits the slowest postnatal growth rate of all mammals, most likely representing an important privilege of evolution promoting brain maturation and cognitive functions. Notably, rapid weight gain in infancy has been linked to an increased risk of asthma [19,20]. Lower birth weight (small for gestational age) was associated with an increased risk of asthma hospitalization in term children [21]. Notably, increased postnatal catch-up growth in small for gestational age infants is associated with increased postnatal mTORC1 signalling and increased childhood asthma risk [22]. Moreover, excessive postnatal protein intake by formula feeding increases weight gain, total body fat mass and increases the risk of childhood obesity [23-25], which has been linked to increased amino acid-mediated mTORC1-signalling [26].

**Figure 1:**
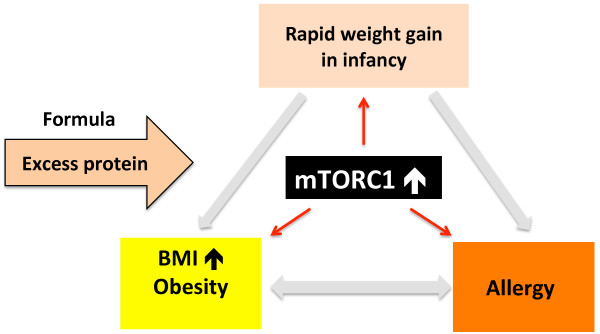
**Association between rapid weight gain in infancy and allergy and obesity development.** The underlying mechanistic pathway that may link these phenomena is exaggerated mTORC1 signalling.

Epidemiological studies clearly confirm the intraindividual association between allergy and obesity [20,27-29]. Prospective cohort studies and two recently published meta-analyses found an association between overweight, especially obesity, and asthma in the appropriate temporal sequence and in a dose–response manner [30]. Children with a pronounced weight gain slope in early life were particularly at risk for asthma within the first 6 years of life. The gain in body mass index (BMI) over time during infancy thus appears to be a more important predictor for asthma in childhood than excess weight at any specific age (Figure 1) [30].

### mTORC1 and T-cell activation

The metabolic demands of T-cells are extraordinary [31]. Naïve T-cells are catabolic and exhibit low levels of mTORC1 activity [32]. However, activation and effector generation for both CD4^+^ and CD8^+^ T-cells results in tremendous metabolic demands and a switch from catabolism to anabolism associated with an increase in mTORC1 activity [32]. The immunosuppressive natural mTOR-inhibitor rapamycin downregulates T-cell anabolism and induces T-cell autophagy thereby suppressing T-cell effector function [32]. The differentiation of helper T-cells is regulated through the selective activation of mTORC1 and mTORC2 [33]. Only mTORC1 integrates environmental cues such as amino acids, energy, and growth factors [32]. Amino acid abundance in the postnatal period by excess protein intake due to artifical formula feeding may thus overstimulate mTORC1 of various immune cells. On the other hand, amino acid deprivation inactivates mTORC1 and T-helper cell activity and induces T-cell anergy [34,35]. Recent evidence confirms that mTORC1 signalling plays a critical role during the early phases of allergic asthma [36,37], consistent with studies showing a role for mTORC1 in early activation and differentiation events of immune cells [33]. Thus, exaggerated mTORC1 signalling by high protein formula feeding may disturb early postnatal mTORC1-mediated immune cell programming.

### Insulin, IGF-1, AKT and regulatory T-cells

**Figure 2:**
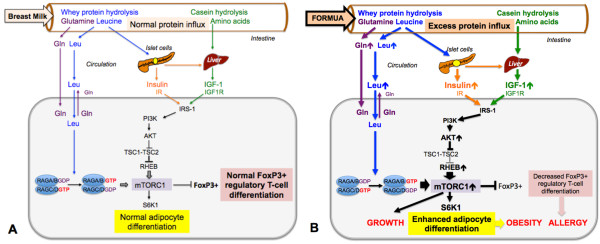
**Comparison of breast milk- and formula-mediated mTORC1 signalling. A**. Human breast milk guarantees the appropriate intestinal influx of milk proteins that after hydrolysis release leucine (Leu) and glutamine (Gln) into the circulation. Whey-derived leucine stimulates insulin secretion, whereas casein-derived amino acids stimulate hepatic IGF-1 synthesis. Insulin and IGF-1 via PI3K activate AKT that attenuates the inhibitory activity of the tuberin complex (TSC1-TSC2) towards RHEB, the GTPase that finally acivates mTORC1. Glutamine enhances cellular leucine uptake that activates the RAG GTPases, the essential step for mTORC1 activation at the lysosomal surface. Physiological mTORC1 activation results in normal activation of the kinase S6K1 that controls adipocyte differentiation and adequate expression of FoxP3^+^ regulatory T-cells. **B**. Excess protein uptake by infant formula feeding enhances plasma levels of leucine, insulin and IGF-1 overactivating mTORC1. Overstimulated S6K1 enhances adipocyte differentiation and inhibits FoxP3 expression, thus promotes growth, obesity and allergy development. Abbreviations: IR: Insulin receptor; IGF-1 insulin-like growth factor-1; IGF1R: IGF-1 receptor; IRS-1: Insulin receptor substrate-1; PI3K: Phosphoinositiol-3 kinase; AKT: Akt kinase (protein kinase B); TSC1: Hamartin; TSC2: Tuberin; RHEB: RAS-homolog enriched in brain; RAG: RAS-related GTP-binding protein; S6K1: Ribosomal protein S6 kinase, 70-KD.

The amino acids leucine, glutamate, and isoleucine are potent insulin secretagogues [38,39]. Excessive uptake of amino acids by formula feeding explains enhanced insulin secretion in comparison to breastfeeding [40-42]. Axelsson et al. [40] determined the urinary C-peptide concentration, a measure of insulin secretion, in breastfed versus formula-fed infants. Whereas breastfeeding exhibited the lowest urinary C-peptide levels (2.2 ± 2.1 nmol/L), feeding low protein formula (1.3 g protein/100 mL) resulted in 32% increased C-peptide levels (2.9 ± 1.9 nmol/L). Notably, high protein formula (1.8 g protein/100 mL) feeding induced 218% elevated urinary C-peptide concentrations (7.0 ± 4.8 nmol/L). Protein-dependent insulinotropic effects of formula feeding have recently been confirmed by a multicentre study of the *European Childhood Obesity Trial Study Group* (Figure 2B) [41].

The insulinotropic amino acid leucine stimulates insulin synthesis and insulin secretion of pancreatic β-cells [38], and activates the translational regulators 4E-BP1 and the kinase S6K1 in an mTORC1-dependent manner [43]. Furthermore, the glutaminolysis pathway plays a pivotal role for insulin secretion. In comparison to breast milk, glutamate, the principal precursor of the glutaminolysis pathway [39], is increased by 60% in lower protein- and 164% in higher protein infant formula [41]. Of key regulatory function for the glutaminolysis pathway is the enzyme glutamate dehydrogenase (GDH), which catalyses the oxidative deamination of glutamate to α-ketoglutarate. The later is a substrate of the Krebs cycle that generates ATP required for insulin secretion. Notably, leucine acts as an allosteric activator of GDH that contributes to leucine sensing involved in the upregulation of mTORC1 [44,45]. The combination of glutamine and leucine, which maximizes the flux through GDH, is most effective in phosphorylation of S6K1 in β-cells [43]. Taken together, leucine and glutamine in a synergistic fashion activate mTORC1 promoting insulin synthesis and secretion of β-cells [46].

The majority of circulating IGF-1 is produced in the liver (Figure 2). Insulin and amino acids independently stimulate hepatic IGF-1 synthesis [47]. Amino acid availability of hepatocytes is essential for IGF-1 gene expression [48]. In rat hepatocytes, amino acid excess increased IGF-1 expression [49]. In ovine hepatocytes, growth hormone and amino acids synergistically enhanced IGF-1 production. Thus, amino acid excess by formula feeding may overstimulate hepatic IGF-1 synthesis. In fact, Socha et al*.*[41] demonstrated that breastfeeding results in much lower total serum IGF-1 levels of 14.1 ng/mL than formula feeding. Infant formula with lower protein content (2.2 g protein/100 kcal) induced IGF-1 serum levels of 34.7 ng/mL (+146%), whereas high protein formula (4.4 g protein/100 kcal) raised IGF-1 serum concentrations to 48.4 ng/mL (+243%) in infants at the age of 6 months. Furthermore, increased plasma levels of insulin and IGF-1 have recently been reported in formula-fed Rhesus monkeys that received increased amounts of protein (1.83 g/dL) in comparison to Rhesus milk (1.16 g/dL) [42].

Taken together, high protein infant formula feeding overstimulates insulin and IGF-1 production, which both stimulate the downstream kinase AKT [50] that finally activates mTORC1 (Figure 2) [7-10]. Intriguingly, AKT-mediated phosphorylation of FoxO transcription factors leads to their nuclear extrusion into the cytoplasm. Notably, a FoxO3a-binding motif is present in the proximal region of the *Foxp3* promoter [51]. Both FoxO1 and FoxO3a exert stimulatory effects on FoxP3 expression, the key transcription factor of regulatory T-cells (Tregs) [51]. There is convincing evidence that enhanced AKT-mTORC1 signalling suppresses *de novo* differentiation of FoxP3^+^ Tregs in the thymus [52,53]. In summary, exaggerated insulin/IGF-1 signalling, a characteristic endocrine feature of recent formula feeding impairs Treg maturation.

### Amino acids, mTORC1, and regulatory T-cells

Effector T-cell differentiation requires the integration of multiple signals. Recent evidence underlines that mTOR signalling dictates the outcome of Treg lineage commitment [54]. Under normal activating conditions, T-cells lacking mTOR differentiate into FoxP3^+^ Tregs [32,54]. Given that essential amino acids are important nutrient signals activating mTORC1 [15-17], it could be expected that amino acid abundance suppresses the differentiation of Tregs. On the other hand, amino acid deprivation should attenuate mTORC1 signalling. Indeed, Cobbold et al. [35] demonstrated that T-cells failed to proliferate in response to antigen, when one or more essential amino acids were limited, which was associated with reduced mTORC1 signalling. Importantly, inhibition of mTORC1 by limiting essential amino acids induced FoxP3 expression [35]. There is convincing evidence that the AKT-mTORC1 signalling axis negatively regulates *de novo* differentiation of CD4^+^FoxP3^+^ Tregs in the thymus [52,53]. Overactivated mTORC1 downregulates the expression of FoxP3, the key transcription factor of Tregs [55]. In nasal polyps, whose association with allergy is still controversial [56], significantly increased infiltration of mTOR-activated inflammatory cells and decreased infiltration of FoxP3^+^ Tregs have been detected [57]. Excess protein intake by formula feeding may thus overstimulate mTORC1 signalling compromising Treg differentiation. On the other hand, mTORC1 activity is necessary to maintain Treg function partly through inhibiting the mTORC2 pathway [58]. In mice, TSC1 has been shown to play an important role in regulating thymic CD4^+^CD25^+^Foxp3^+^ nTreg-cell development via a rapamycin-resistant and mTORC2-dependent signaling pathway [59].

### Regulatory T-cell deficiency in atopy

Tregs are essential for the maintenance of immune homeostasis and inhibit the differentiation of Th2-cells, suppress the production of IL-4, block the migration of effector T-cells into inflamed tissue, suppress the production of immunoglobulin E (IgE), induce IgG4 in B cells and limit Th17-mediated inflammation [60,61]. Tregs have been identified as key players that prevent allergy development and are the target of immune therapy in the treatment of allergic diseases [61-64]. Tregs are decreased in plasma and sputum of asthma patients and plasma of children with asthma, atopic dermatitis and food allergy [65,66]. In atopic children, Treg deficiency was linearly related to increased IgE serum levels [65].

### IL-4 increase in formula-fed Rhesus monkeys

Excessive protein intake with exaggerated mTORC1 signalling during a most vulnerable period of postnatal Treg maturation may result in deficient Treg-mediated suppression of Th2-cell differentiation increasing the production of IL-4, the signature cytokine of Th2-driven IgE responses. Intriguingly, formula-fed Rhesus monkeys, that received excess amounts of protein (1.83 g/dL) in comparison to Rhesus milk (1.16 g/dL) exhibited significantly increased serum levels of IL-4 associated with accelerated growth in the first month of life [42].

### Bifidobacteria and inducible regulatory T-cells

Tregs are not only generated in the thymus (nTregs), but are also induced in the periphery (iTregs) such as the intestine [58]. Formula feeding in comparison to breastfeeding compromises the development of the physiological gut microbiome. In breastfed Rhesus infants *Bifidobacteria* and *Lactobacillus* predominated, whereas in formula-fed infants *Ruminococcus* was predominant [42]. Breastfed human infants harbor a fecal microbiota more than twice increased in *Bifidobacterium* numbers compared to formula-fed infants [67]. After formula feeding, *Atopobium* was found in significant counts and the numbers of *Bifidobacteria* dropped followed by increasing numbers of *Bacteroides* population [67]. Remarkably, breast milk is a natural source of bifidobacteria, which play an important role for infant gut development and maturation [68,69]. Complex oligosaccharides in breast milk support the establishment of bifidobacteria in the neonatal gut, which finally stimulate iTreg development [70]. In fact, oral consumption of *Bifidobacterium infantis* 35624 is associated with enhanced FoxP3 expression in human peripheral blood cells pointing to the immune-stimulatory effect of bifidobacteria on FoxP3^+^ iTreg induction [71,72]. It has recently been demonstrated that bifidobacteria stimulate transforming growth factor-β (TGFβ), which contributes to Treg differentiation [73]. Taken together, the probiotic effect of bifidobacteria in the prevention of atopic diseases appears to be related to their capability to generate FoxP3^+^ iTregs. In contrast, infant formula, that lacks bifidobacteria, may impair appropriate intestinal generation of FoxP3^+^ iTregs.

### Immune-regulating microRNA deficiency in infant formula

Early-life consumption of unboiled cow’s milk has been characterized in several studies of human infants to be a protective factor for the development of atopy [74-83]. Indeed, farm milk exposure has been associated with increased numbers of CD4^+^CD25^+^FoxP3^+^ Tregs, lower atopic sensitization and asthma in 4.5-year-old children [84].

The heat-sensitive atopy-preventive factor in fresh unboiled cow’s milk has not yet been identified. Nevertheless, human and bovine milk contain substantial amounts of exosomal microRNAs, which have been postulated to be involved in postnatal immune regulation [85-90]. Milk microRNAs are transported by membranous microvesicles, called exosomes that play a pivotal role for horizontal microRNA transfer [91]. Raposo et al. [92] provided first evidence for exosome-mediated immune cell communication. Unidirectional transfer of microRNA-loaded exosomes from T-cells to antigen-presenting cells has recently been confirmed [93]. For immune cell-cell interactions exosome transport exchanging genetic messages over distances has been appreciated [94,95]. Human and bovine milk contain high amounts of exosomal microRNA-155 [86,87,96,97]. Admyre et al. [88] showed that incubation of human peripheral blood mononuclear cells with isolated human milk exosomes increased the number of CD4^+^CD25^+^FoxP3^+^ Tregs in a dose-dependent manner. Substantial evidence underlines that the ancient immune-regulatory microRNA-155 is required for the development of Tregs [98]. Notably, microRNA-155-deficient mice have reduced numbers of Tregs both in the thymus and in the periphery [98]. FoxP3 binds to the promoter of *bic*, the gene encoding microRNA-155 [99-101]. T-cell receptor (TCR) and Notch signalling upregulate the IL-2R α-chain (CD25), rendering thymocytes receptive to subsequent cytokine signals that foster their development into fully functional FoxP3^+^ Tregs [102-104]. IL-2 is capable of transducing signals in CD4^+^FoxP3^+^ Tregs as determined by phosphorylation of signal transducer and activator of transcription 5 (STAT5) [104]. Deletion of microRNA-155 results in limited IL-2/STAT5 signalling, which reduced Treg numbers [105]. Remarkably, microRNA-155 enhances FoxP3 expression by targeting suppressor of cytokine signalling 1 (SOCS1), an important negative regulator of IL-2R/STAT5 signalling [105].

Boiling of farm milk abolishes the atopy-preventive effect of cow’s milk [74-83]. Boiling of cow’s milk degrades milk-derived bioactive microRNAs [90]. It has been shown that exosome membrane integrity is essential for the uptake of milk microRNAs into cultured cells [96]. The boiling process may disrupt the exosome lipid bilayer thus exposing the microRNA cargo to rapid RNase-mediated degradation. These observations support the recent concept that milk’s exosomal microRNAs may be involved in the maturation of Tregs and provide a potential signalling network of fresh milk that controls adequate maturation of Tregs preventing allergic immune deviations [106]. Indeed, exosomes have been detected in the intestine [107], and in the human and murine thymus [108,109], where they induce Tregs [108]. It is thus conceivable that during the postnatal period, a time with higher intestinal permeability, immune-regulating exosomal microRNAs may pass the intestinal permeability barrier and traffic to the thymus or peripheral lympoid organs to promote Treg maturation [110]. Whereas raw unprocessed cow’s milk contains the highest amounts of bioactive microRNAs of all known body fluids, pasteurized milk contains much lower levels and milk powder commonly used for infant formula production only exhibits trace amounts of RNAs [86,87]. The absence of bioactive microRNA-155 in infant formula may lead to inappropriate intestinal or thymic Treg maturation providing a further argument for the allergy-promoting effect of formula feeding and for the atopy-preventive effect of raw cow’s milk consumption early in life [106].

### Breast milk n-3 polyunsaturated fatty acids and Treg maturation

Maternal long-chain polyunsaturated fatty acid (LC-PUFA) intake impacts their delivery to the infant either via the placenta or breast milk [111]. Supplementation of pregnant women with n-3 PUFAs has been reported in some studies to decrease sensitization to common food allergens and to lower the prevalence and severity of atopic dermatitis in the first year of life [112,113]. There appears to be an effect of n-3 PUFAs in early life programming of the immune system [114]. In a murine model of cow’s milk allergy it could be demonstrated that allergy prevention by n-3 LC-PUFA was mediated by the induction of Tregs [115,116]. Yasuda et al. [117] recently demonstrated that fatty acids have a profound impact on mTORC1 regulation. Whereas the saturated fatty acid palmitate activated mTORC1 by enhancing the translocation of mTORC1 to the lysosome, the unsaturated n-3 fatty acid eicosapentaenoic acid inhibited saturated fatty acid-induced translocation of mTORC1 to the lysosome and its subsequent activation [118]. n-3-fatty acid-mediated attenuation of mTORC1 activity may thus increase the maturation and function of Tregs. Breast milk-derived n-3 LC-PUFAs may represent another milk-derived control layer regulating Treg maturation.

## Conclusions

Milk is the species-specific, genetically highly conserved end product of lactation that ensures the appropriate magnitude of mTORC1 activity of the milk recipient, which plays a fundamental role in adipocyte and Treg maturation [26,106,110]. The vector system milk exhibits a *hardware*, primarily represented by essential branched-chain amino acids and glutamine for appropriate mTORC1-dependent maturation of FoxP3^+^ Tregs [106,110]. Milk’s *software* appears to be represented by the delivery of exosomal immune-regulatory microRNAs [85-90]. Especially the ancient microRNA-155 appears to play a crucial role for FoxP3 expression [98,105]. The relevance of iTregs to the development of atopy in the first year of life is less clear. However, the *probiotic system* of milk featured by milk-derived bifidobacteria regulates the appropriate development of the infant’s gut microbiome by providing bifidobacteria as well as bacterial nursing factors such as oligosaccharides of human milk [118-121]. The appropriate composition of milk fatty acids may have further impacts on mTORC1-mediated Treg differentiation [115-117].

Thus, at least five interacting signalling networks of milk control the induction of FoxP3 and Treg maturation: 1) amino acid-mediated regulation of mTORC1, 2) milk exosomal microRNA-mediated FoxP3 expression, 3) milk-derived bifidobacteria and their growth-promoting glycobiome, 4) fatty acid-mediated modulation of mTORC1-dependent Treg maturation, and 5) the least characterized role of milk-derived stem cells with the potential of multilineage differentiation [122,123] (Table 1).

**Table 1:** **Effectors of postnatal feeding regulating FoxP3**^**+ **^**Treg differentiation**

**Effectors**	**Mechanisms of FoxP3-induction**
**Amount of protein influx** (Excessive by formula feeding)	Protein and amino acids regulate the magnitude of AKT- and mTORC1-activity that controls the expression of FoxP3.
**Exosomal microRNA** (Absent in artificial formula)	Exosomal microRNA-155 enhances the expression of FoxP3, which promotes microRNA-155 expression.
**Bifidobacteria** (Absent in artificial formula)	Breast milk delivers bifidobacteria and milk-derived oligosaccharides that promote bacterial growth in the gut. Bifidobacteria induce intestinal generation of FoxP3^+^ iTregs.
**Polyunsaturated fatty acids** (Not adequately provided by formula feeding)	n-3-Polyunsaturated fatty acids inhibit mTORC1 activation, thus promote FoxP3 expression, whereas saturated fatty acids activate mTORC1, thus attenuate FoxP3 expression.

Mammalian lactation exhibits a highly sophisticated growth-promoting, immune-regulating, and adipocyte differentiating signalling network donated by mammary glands secretory end product milk, which is obviously not *just food* as misinterpreted in the early 1930’s [3,4].At the molecular level, both the development of allergy and childhood obesity can be traced back to exaggerated mTORC1 signalling during a vulnerable window of postnatal programming. Accelerated postnatal growth and weight gain just reflect overstimulated mTORC1 signalling during a sensitive period of human nutrition, a period of life, in which the nutritional route is used for lifelong metabolic and immunologic programming (Figure 1).

The vigorous change from this evolutionarily developed and genetically highly conserved system of breastfeeding to an artificial programming procedure apparently represents the most serious error of modern medicine laying the foundation for the worldwide epidemics of allergy and obesity. This man-made disturbance of early mTORC1-programming explains the comorbidity of allergy and obesity.

It has to be kept in mind that not only the postnatal period plays a fundamental role in metabolic and immunological programming but also fetal development [124]. Maternal obesity has been related to fetal overgrowth and high birth weight. The later has been linked to obesity and type 2-diabetes later in life [125,126]. In contrast, rapid catch-up growth after intraunterine growth restriction has been associated with the development of asthma [21,127]. Placental nutrient transport is controlled by insulin/IGF-1 and mTORC1 signalling and is upregulated in fetal overgrowth [128,129]. Notably, regular cow’s milk consumption increases both insulin/IGF-1- and mTORC1 signalling [110]. It is thus not surprising that milk consumption during pregnancy increased fetal growth, infant size at birth and birth weight [130,131]. In terms of evolutionary biology, regular cow’s milk consumption is a very recent behavorial change, which may have adverse long-term health effects in humans [132]. During the last century, due to the implementation of widespread cooling technology milk and other dairy products became alvailable on a large scale and cow’s milk-based artificial infant feeding have been introduced into human biology. Both, cow’s milk consumption during pregnancy and artificial infant feeding practices may contribute to aberrant perinatal metabolic and immunologic programming. Future research has to clarify which periods of perinatal life are most vulnerbale for aberrations of nutritional mTORC1-mediated programming. Rowe et al. [133] demonstrated that priming of Th2 responses associated with persistent house dust mite (HDM)-IgE production occurs entirely postnatally, as HDM reactivity in cord blood seems nonspecific and was unrelated to subsequent development of allergen-specific Th2 memory or IgE. These observations underline the importance of the postnatal period, which physiologically depends on the programming effects of breastfeeding. Only breastfeeding guarantees the species-specific mTORC1-signalling axis controlled by the human lactation genome that developed during millions of years of mammalian evolution. After one century of formula feeding it is time to remember the words of Dr Truby King who stated in 1913 in his book *Feeding and Care of the Baby* that the natural food direct from the mother’s breast is the child’s birth right [3,134].

## Abbreviations

AKT: Akt kinase (protein kinase B); BMI: Body mass index; 4-EBP1: Eukaryotic translation initiation factor 4E-binding protein 1; FoxP3: Forkhead box P3 (scurfin); IgE: Immunoglobulin E; IGF-1: Insulin-like growth factor-1; IGF1R: IGF-1 receptor; IL-4: Interleukin 4; IR: Insulin receptor; IRS-1: Insulin receptor substrate-1; LC: Long-chain; mTORC1: Mechanistic target of rapamycin complex 1; PI3K: Phosphoinositol-3 kinase; PUFA: Polyunsaturated fatty acid; RAG: RAS-related GTP-binding protein; RHEB: RAS-homolog enriched in brain; S6K1: Ribosomal protein S6 kinase, 70-KD (RPS6KB1); SOCS1: Suppressor of cytokine signalling 1; STAT5: Signal transducer and activator of transcription 5; TCR: T-cell receptor; TGFβ: Transforming growth factor-beta; Treg: regulatory T-cell; TSC1: Hamartin; TSC2: Tuberin.

## Competing interests

The author declared that he has no competing interests.
